# Developing initial programme theories for a realist synthesis on digital clinical consultations in maternity care: contributions from stakeholder involvement

**DOI:** 10.1177/17449871241226911

**Published:** 2024-03-08

**Authors:** Catrin Evans, Georgia Clancy, Kerry Evans, Andrew Booth, Benash Nazmeen, Stephen Timmons, Candice Sunney, Mark Clowes, Nia Wyn Jones, Helen Spiby

**Affiliations:** Professor in Evidence Based Healthcare, School of Health Sciences, University of Nottingham, Nottingham, UK; Research Fellow, School of Health Sciences, University of Nottingham, Nottingham, UK; Senior Clinical Academic Midwife and Associate Professor, School of Health Sciences, University of Nottingham, Nottingham, UK; Professor in Evidence Synthesis, ScHARR, University of Sheffield, Sheffield, UK; Assistant Professor, School of Allied Health Professionals and Midwifery, University of Bradford, Bradford, UK; Professor of Health Services Management, Nottingham University Business School, University of Nottingham, Nottingham, UK; Lay Researcher, Nottingham Maternity Research Network, Nottingham, UK; Information Scientist, ScHARR, University of Sheffield, Sheffield, UK; Clinical Associate Professor, School of Medicine, University of Nottingham, Sheffield, UK; Professor of Midwifery, School of Health Sciences, University of Nottingham, Nottingham, UK

**Keywords:** digital health, maternity, midwife, nurse, realist synthesis, stakeholder involvement, tele-health

## Abstract

**Background:**

The COVID pandemic prompted an increase in the use of digital clinical consultations (telephone or video calls) within midwifery and nursing care. This paper reports on a realist review project related to maternity care that seeks to illuminate for whom such consultations can safely and acceptably be used, how, for what purposes and in what contexts.

**Aims:**

This paper addresses the first phase of a realist enquiry – initial programme theory development – focusing particularly on the role of stakeholder involvement (including digital transformation leaders, midwives, obstetricians, service users and community organisations).

**Methods:**

Three sub-stages of initial programme theory development are described highlighting the contribution of stakeholder groups to each stage: (i) consultation to focus the review question, (ii) focused searching and (iii) further consultation.

**Results:**

Realist literature searching strategies yielded limited theory-rich evidence on digital consultations. Stakeholders provided essential additional contributions resulting in the development of 13 initial programme theories and a conceptual framework.

**Conclusions:**

More research on the implementation of virtual midwifery/nursing consultations is needed. Nursing/midwifery digital researchers should involve stakeholders to help shape research priorities, deepen contextual understanding and sense-check emerging findings.

## Introduction

Health services in the UK are currently implementing large-scale digital transformation programmes. As frontline providers of care, nurses and midwives have a critical role to play in leading research and service innovations related to digital health (Department for Health and Social Care, 2022; National Maternity Review, 2016; NHS Digital, 2021; NHS England Maternity Transformation Programme, 2016). Accordingly, there is an ongoing need for midwives and nurses to develop competencies in utilising and researching new technologies (Clarke-Darrington et al., 2023).

One component of digital transformation relates to changes in consultation modality – in which face-to-face healthcare interactions are replaced or supplemented with digital, remote or virtual means (telephone or video calls). This paper reports on an ongoing evidence synthesis project – focused on the maternity setting – that uses a realist methodology to explore how digital clinical consultations can be implemented in a clinically safe, appropriate and acceptable way (Evans et al., 2022). Within the project, the term ‘digital clinical consultation’ (hereafter referred to as DC-CON) is defined as follows: *Synchronous telephone or video consultations involving direct interaction between a service user and a maternity healthcare professional. It has two-way functionality and can be initiated by either party. It may be linked to, or complemented by, other digital technologies within the maternity care pathways.*

Pre-pandemic, there was a small but growing trend of innovations using DC-CON within maternity care (e.g. for breastfeeding or smoking cessation support, or creating ‘hybrid’ models of antenatal care). Research suggests that such innovations can be feasible, safe, effective and acceptable (Butler Tobah et al., 2019; DeNicola et al., 2020; Lavender et al., 2013; Marko et al., 2016; van den Heuvel et al., 2018). This pre-COVID evidence however, consists of relatively small-scale studies, undertaken with well-resourced interventions and carefully controlled samples in which participants were offered choices and alternatives regarding their participation.

By contrast, the COVID-19 pandemic forced a wide-spread implementation of DC-CON across the whole maternity system. Guidance was produced on the use of remote consultations (Royal College of Midwives, 2021a, 2021b; Royal College of Midwives and Royal College of Obstetricians & Gynaecologists, 2020), but studies on maternity care during the pandemic, perhaps unsurprisingly, provide a more mixed picture, with both positive and negative experiences reported amongst women and midwives (Flaherty et al., 2022; Hinton et al., 2022; Jeganathan et al., 2020; Karavadra et al., 2020). In addition, several authors have raised concerns about the potential for DC-CON to exacerbate inequalities, but there is little evidence about which groups may be most affected or the specific pathways involved (Hinton et al., 2021; Renfrew et al., 2020). It is currently unclear therefore how, for whom or in what contexts, DC-CON should be used as part of routine midwifery care. Our realist synthesis seeks to address some of these uncertainties. The project is led by nurses and midwives, working within a wider multi-professional and multi-disciplinary team.

Realist approaches are increasingly being used in health services research (see Supplemental File S1 that explains realist terminology). Instead of evaluating the success of an intervention, realist inquiry seeks to illuminate causal relationships (expressed as ‘programme theories’) between intersecting intervention components, contexts and outcomes. (Pawson and Tilley, 1997). For example, in DC-CON, different types of interventions (e.g. a video call or a phone call) supply resources into a context that can produce diverse reactions and responses (referred to as ‘mechanisms’) from women and midwives. In realist approaches, programme theories are often expressed using a C–M–O (context–mechanism–outcome) heuristic to provide an understanding of ‘how things work’. By developing theoretical understandings of intervention implementation, realist research provides practitioners with evidence to adapt intervention components across systems where complexity and diversity are the norm (Rycroft-Malone et al., 2012).

Following established guidance (Wong et al., 2013), realist reviews adopt an iterative 3 Phase process that involves: (1) exploratory searches and consultations to construct a set of initial programme theories (IPTs), (2) a comprehensive search of relevant evidence to test and elaborate the IPTs and (3) a process of theory refinement through more focused literature searches or primary data collection.

## Aim

The aim of this paper is to describe the first Phase of the project, in which IPTs were developed. To date, there is limited guidance on this key step of the review process, especially for theorising large complex initiatives (Shearn et al., 2017). In addition, although it is widely recommended to involve service users and other relevant stakeholders (e.g. staff) within realist review processes, there has been limited guidance on how this can best be achieved, what impact it has, or its role specifically in relation to the first phase of IPT development (Abrams et al., 2021). This paper focuses particularly on the importance of involving stakeholders in the initial Phase of the project, exploring their contribution and reflecting on the strategies required. Because of this emphasis, some of the more technical details of the IPT development are reported in Supplemental Files. We hope the methodological insights as well as the IPTs themselves will be helpful for nurses/midwives undertaking similar enquiries across different clinical areas.

## Project structure

**Figure 1.**
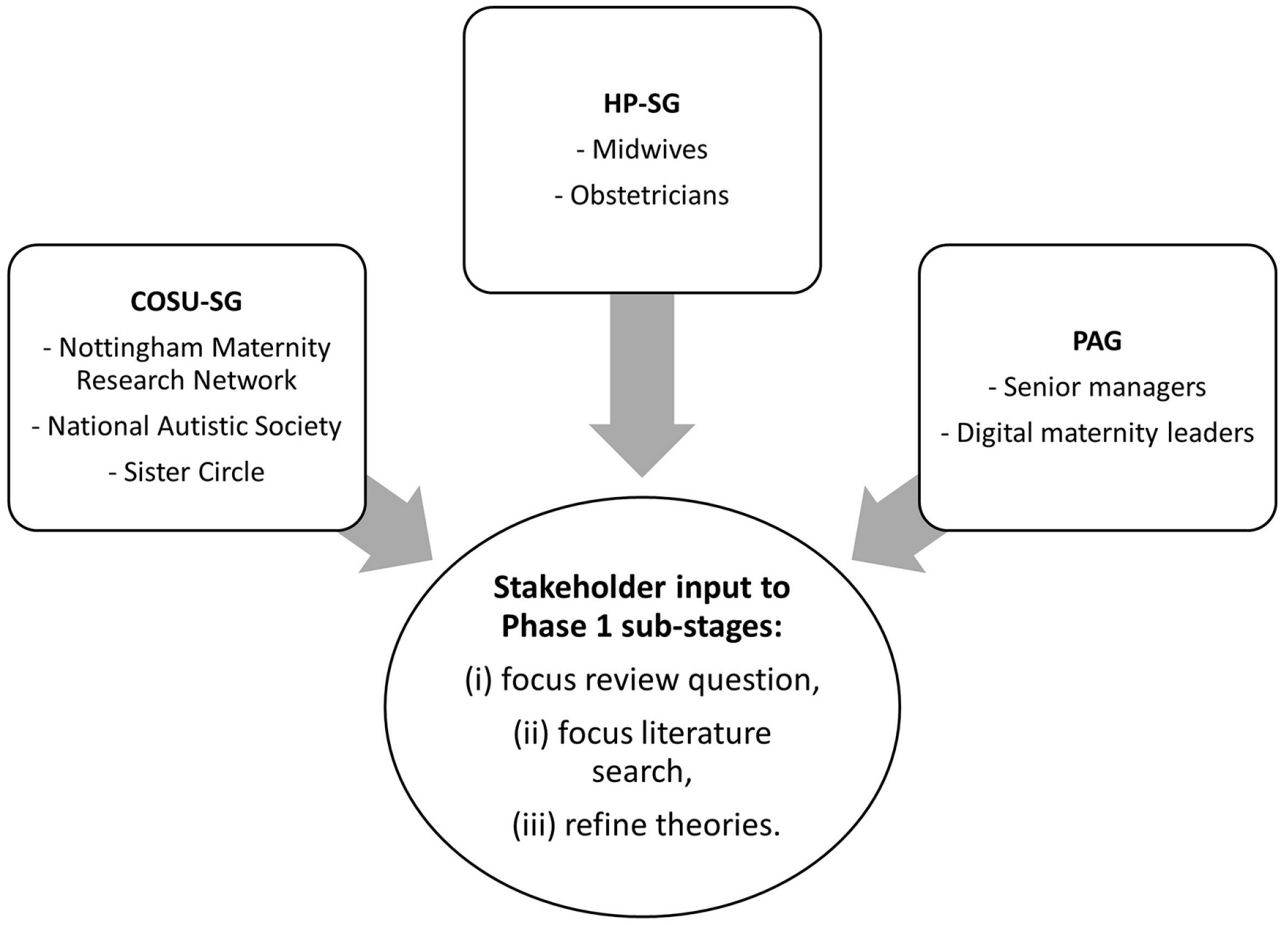
Stakeholder groups involved in the project.

The project originated from a midwifery-led research priority setting exercise undertaken in Nottingham/Nottinghamshire in 2020 involving several organisations, including a maternity research public involvement group – the ‘Nottingham Maternity Research Network’ (NMRN; Evans et al., 2021). A service user representative from the NMRN subsequently became a co-applicant on the funding bid, thus embedding service user engagement into the project from the outset. The team wanted to ensure the project was informed by the views of relevant stakeholders, and that these should, ideally, represent a diversity of experiences. Two stakeholder groups were formed: a ‘Community Organisation and Service User Stakeholder Group’ (COSU-SG) and a ‘Health Professional Stakeholder Group’ (HP-SG). The COSU-SG included members from the NMRN, but it was also considered important to include perspectives relating to neuro-diversity (as social communication differences may have been particularly salient for DC-CON), and to be able to reflect experiences of women from a wider range of geographical locations, ethnic and socio-economic backgrounds. These perspectives were sought by inviting the participation of maternity advocates from the National Autistic Society and from ‘Sister Circle’ (previously known as ‘Women and Family Health Services’) – a London based charity working with women experiencing complex social disadvantage, including those with English as a second language. The COSU-SG included 13 members in total, of whom 5 had been pregnant during the pandemic; others had recent experience of maternity care or of directly supporting pregnant women (as peer support advocates) through their entire maternity journeys. The HP-SG comprised 26 midwives and obstetricians from across the maternity system, recruited via social media and specialist interest groups (see Supplemental File S2 for further detail of the groups). A Project Advisory Group (PAG) comprising senior managers and leaders in digital maternity provided additional high-level insights/linkages with policy and commissioning and horizon scanning. See Figure 1 for a depiction of stakeholder involvement in the project.

## Methods

**Figure 2.**
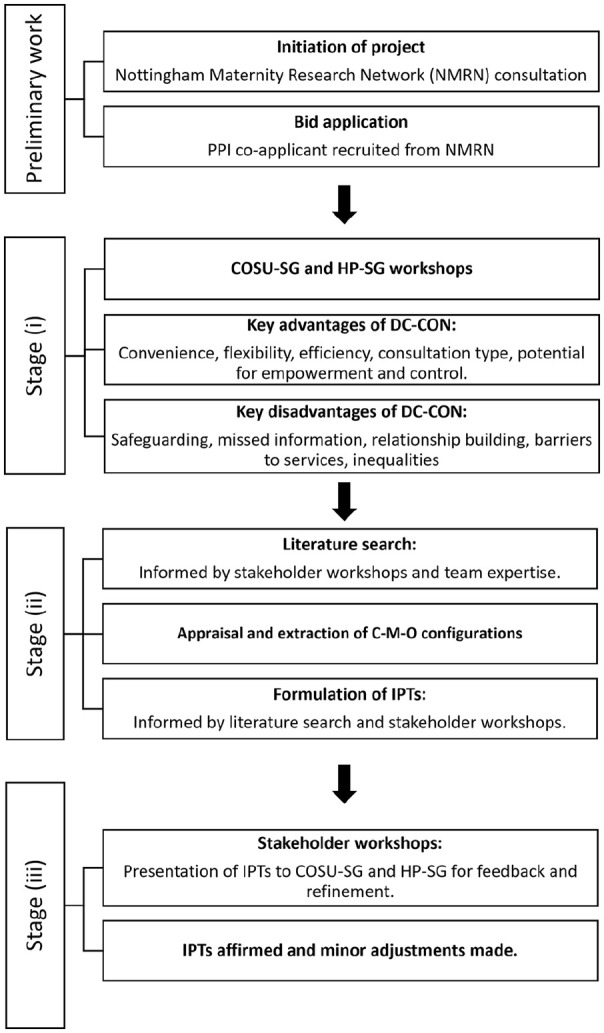
Project process flowchart.

The first (IPT development) Phase of our realist review had three sub-stages: (i) consultation to refine and focus the review question; (ii) searching for theory-rich evidence which was analysed to construct project-specific IPTs and (iii) further consultation. Insights from the COSU-SG, HP-SG and PAG were involved in all of these sub-stages. The endpoint of the first Phase was an agreed set of IPTs – to be taken forward for evaluation and refinement in Phases 2 and 3 of the review (Wong et al., 2013). See Figure 2 for a flowchart of the project process.

### Stage I

The project started with workshops for each stakeholder group in which participants were asked about their experiences and views regarding the key issues that the project team should attend to. For both groups, perceived benefits of DC-CON included convenience, flexibility and efficiency, especially for consultations perceived to be ‘routine’ or not requiring physical examination. The potential for empowerment, control and reassurance were identified as possible mechanisms underpinning a positive experience of DC-CON. However, the workshops also highlighted the need to problematise assumptions around concepts such as ‘routine’ or ‘non-complex’ care, identifying that responses to remote care could be highly variable and influenced by individualised preferences, regardless of the reason for consulting. A strong emphasis was placed on the need for personalisation and choice in understanding the appropriate use of DC-CON – mirroring the emphasis that is given to these principles within midwifery practice and national NHS and maternity policy (National Maternity Review, 2016; NHS England, 2019, 2021).

Key disadvantages included concerns around safeguarding, missing important information/issues, difficulty in building meaningful relationships and amplification of existing inequalities. In particular, concerns were expressed about additional barriers to service access and utilisation for women with poor access to the internet (or to technology) or with other communication challenges (e.g. English as a second language). Other barriers included a standardised approach to implementation (making it difficult to offer choice or flexibility to service users), lack of staff confidence/competence in using new systems and processes, problems with technology and inter-operability of systems and the need to undertake ‘workarounds’ creating additional unseen workload. Key outcomes were service user/staff experience, workload, equity, access, clinical and cost-effectiveness and safety.

Participants in both groups strongly emphasised the need to consider DC-CON in terms of its appropriateness and impact with respect to different groups of women. Both stakeholder groups stressed the importance of inclusion, attention to diversity and the danger of digital consultations exacerbating inequalities. It was important, therefore, for the process of IPT development to take into account features of context that might link to inequality and inclusion. These concerns were incorporated within a sampling process used for evidence retrieval in Stage II.

### Stage II

The next stage of refining the review focus and IPT development comprised a focused search of the literature in order to identify papers and other sources of evidence with sufficient detail and richness to contribute to theory building (Booth et al., 2018). The insights from the stakeholder (COSU-SG, HP-SG and PAG) workshops in stage I (as well as expertise from within the team) were utilised to construct an initial table listing potential contexts, mechanisms and outcomes (CMOs). This exercise helped ensure that we tried to find relevant literature relating to the dimensions that had been deemed important. Accordingly, contexts were defined in relation to population groups (e.g. different groups of women with different social, economic or clinical issues); settings (e.g. rural or urban), stages of the maternity pathway, DC-CON modality and associated technologies (e.g. phone or video, linkage to record systems) and organisation of care. Mechanisms included the identified concepts such as empowerment, convenience, digital capacity and inclusion and outcomes included a focus on equity (in addition to others such as safety, clinical outcomes and satisfaction).

Initial searches were constructed around database indexing terms and synonyms for the three facets of: (i) remote care, (ii) maternity and (iii) models/frameworks. The search utilised an established approach to searching for theory-rich papers (Booth and Carroll, 2015) and was implemented across three databases (Medline, CINAHL, Scopus). The search was limited to studies from 2010 onwards in order to reflect a contemporary picture of technology use. The results revealed a limited number of theory-rich maternity papers. As a result, we implemented a second search of potentially comparable non-maternity clinical areas (primary care, diabetes, hypertension) and pursued a range of supplementary and grey literature search strategies – see Supplemental File S3 for the search strategy. This initial realist search process therefore needed to draw upon many diverse sources of evidence (within but also outside of the maternity context) and yielded a total of 2638 records (Booth et al., 2018).

Study screening and selection aimed to identify what has been referred to as ‘key informant’ papers (rather than to undertake a comprehensive search of empirical evidence which occurs in Phase 2 of a realist review; Jagosh, 2022). Such papers are defined as: *‘papers that have high relevance to the realist synthesis. . . . . . .the framing of the research and the research questions are highly matched to the review questions, the empirical findings are clearly described and there is a rich description of the process and context that can greatly advance the theoretical output of the review’* (Jagosh, 2022: 1). In addition to using criteria of relevance and richness, we also adopted a purposive sampling approach to study selection. Purposive sampling helped to keep this phase of the review manageable (Ames et al., 2019), but more importantly, it also provided a way of addressing the priorities identified by our stakeholders. The initial tabulated list of CMOs was modified into a sampling framework based upon maximum variation sampling in terms of potential groups of women and settings taking care to ensure that all areas identified as stakeholder priorities were included. See Supplemental File S4 for the final list of papers reviewed (*n* = 49), grouped according to the framework.

This phase of a realist review also includes consideration of potentially relevant mid-range theories – defined as ‘*theories that lie between the working hypotheses that evolve in abundance during day-to-day research and the all-encompassing speculations comprising a master conceptual scheme*’ (Kislov et al., 2019: 4) and conceptual frameworks. We identified one framework (Greenhalgh et al., 2021) – Planning and Evaluating Remote Consultation Services (PERCS) and three mid-range theories: Candidacy (Dixon-Woods et al., 2006), Burden of Treatment (May et al., 2014) and Normalisation Process Theory (May et al., 2007). These theories were deemed potentially relevant as they have been utilised in papers across a range of healthcare settings demonstrating transferable insights. Furthermore, they had direct relevance to the review’s focus on remote consultation implementation processes and had the potential to address concerns around equity and inclusion which had been identified by our stakeholders as key priorities for the review (Hinton et al., 2023).

Each included document was reviewed to elucidate how ‘best practice’ in DC-CON was being conceptualised, what the key outcomes were and to identify potential mechanisms through which consultations are purported to work in relation to different contextual configurations. For each paper and theory, an appraisal/extraction form was completed (see Supplemental Files S5 and S6), which included a prompt to facilitate abductive thinking (inferring plausible explanations based on available evidence). Using an ‘*If-Then-Leading To*’ heuristic, where possible, data were configured into CMO propositions (Jagosh et al., 2014). This resulted in 142 CMO configurations, documented in an Excel spreadsheet.

The next stage was to consolidate and organise these CMO statements into IPTs. This was done using three iterative processes. The first process used thematic analysis techniques to group the CMOs into those that related to women/service users and those that related to healthcare professionals. The CMOs were then reviewed, compared and contrasted to consolidate and differentiate the propositions based on areas of commonality, overlap and difference. The second process involved drawing upon insights from the mid-range theories to help clarify mechanisms and to develop a more abstract and analytical understanding of phenomena. The third process was to construct a theoretically informed conceptual framework to help provide an analytical structure for the wide range of contexts that were identified as important. The aforementioned PERCS conceptual framework was identified as highly suitable for this purpose (Greenhalgh et al., 2021). PERCS is based on a large evidence base for implementation of remote consultations in a range of healthcare settings that spans the pre-COVID time period, but also takes into account lessons learned from studies and experiences during the pandemic. Importantly, it conceptualises digital consultations from a complex systems perspective in which all aspects, actors and contexts need to be considered in order to develop a holistic view of implementation processes, capable of taking into account the dynamic inter-dependencies and interactions occurring between the different parts of the system and at different levels of social structure (e.g. individual, organisation, society). As such, we felt it would be able to capture the concerns for equity, inclusion and choice as emphasised by our stakeholder groups. In addition, by aligning the review with an existing framework, we hope that its findings will be more transferable.

### Stage III

Using the processes above, the CMOs were consolidated into 13 IPTs. These were then presented to the two stakeholder groups in a second round of consultative workshops. The groups affirmed the IPTs but suggested minor changes in emphasis to some of them (such as ‘tweaking’ the language to capture the potential diversity of experience). The HP-SG clarified that a strong motivator for staff to use remote consultations would be feedback that women were satisfied with this approach. The COSU-SG emphasised the potential need to re-visit and continually evaluate individual preferences around digital consultations depending upon women’s changing circumstances during their pregnancy journeys.

## Results: Phase 1 initial programme theories

**Figure 3.**
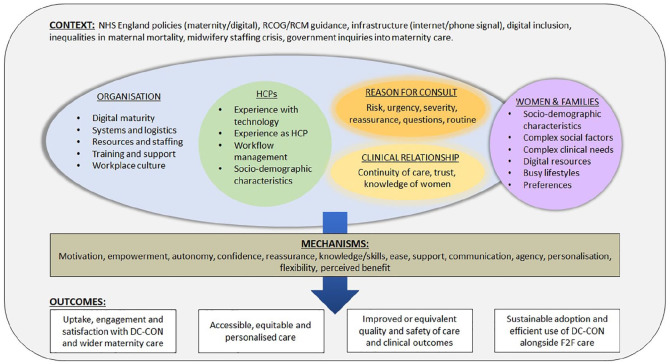
Modified conceptual framework. Note. Please refer to the online version of the article to view the table in colour.

The Phase 1 IPTs are summarised in Figure 3 within a maternity-adapted PERCS conceptual framework. The 13 full CMO propositions are detailed in Supplemental File S7. Overall, the IPTs tentatively propose that remote consultation can be safe and acceptable to stakeholders if implementation processes are able to ensure equity of access, personalisation and professional autonomy. Key mechanisms that support implementation for women and families are proposed to include informed choice, sense of control and empowerment, personalisation, knowledge, motivation, sense of entitlement, ease of use, fit with women’s preferences and lifestyles, reduced treatment burden, reassurance, sense of connection, communication and participation. Underlying contextual conditions for women are proposed to include access to (material, social and digital) resources, capacity and a flexible system that enables information sharing and can adapt to women’s needs and preferences. Key implementation mechanisms for staff include perceived benefit, convenience, motivation, knowledge/skills, perceived support, confidence, professional autonomy, ability to personalise care and develop relationships and communication. Underlying contextual conditions for staff are proposed to include provision of clinical guidance, resources, infrastructure and integration with record systems as part of a workplace culture that provides support and training.

## Discussion

Within the context of a realist review, the IPT development phase described in this paper demonstrates the ways in which this Phase can involve, and be informed by, stakeholder views and experiences alongside other approaches. In addition, our experience shows that for topics that have not yet been heavily theorised (as is the case for DC-CON in maternity settings), it is possible to draw upon wider sources of evidence (e.g. mid-range theories and insights from other comparable settings) to help the IPT development process. The insights from our stakeholder groups (combined with these other diverse evidence sources) enabled us to construct a maternity-focused conceptual framework and IPTs.

The stakeholder workshops were designed to be inclusive and informal. The workshops were held online, enabling busy women and practitioners to easily participate from across the country, increasing the number of people eligible to take part and capturing potential geographical-based differences in experiences and services. The COSU-G stakeholders also received payment for taking part, in appreciation of their time, contribution and any costs they had incurred (e.g. mobile data). The HP-SG and COSU-SG workshops were held separately as we felt this format would create more relaxed environments and, within the time available, enable as many people as possible to share their views, reflecting their different positionalities (as staff or service users). Later in the project, we aim to bring the two groups together to prompt dialogue and debate. Each workshop began with a short presentation on the project and a simple explanation of the realist approach. Following each meeting, group members were sent a plain English summary of the key points and were invited to send in further ideas and comments (one participant did this as she felt more able to describe her experiences in writing). The first workshop had involved open-ended sharing which was relatively straightforward to facilitate. The second workshop however was more challenging as we needed to present complex IPTs in a way that would be accessible for a public audience. Hence, for the COSU-SG, we created a series of scenarios that embedded the IPTs and invited discussion around these (see Table 1 for one example and Supplemental File S8 for other examples). This approach appeared to work very well.

**Table 1.** Example of a scenario for initial programme theory discussions.

**Scenario 1** Jade works part-time, with a toddler and a baby on the way. She has an upcoming appointment to discuss her birth plan with her midwife at the birth centre. However, Jade’s car is currently at the garage having work done and the birth centre is in the next town. This means Jade would have to take three different buses, with her toddler, in order to attend the appointment. The journey will also take much longer on the bus so she might have to take time off work. Concerned about this, Jade rang the birth centre where the staff suggested that they change her appointment to a telephone consultation. Questions for the Group • How do you think Jade would feel about the suggestion to change her face-to-face appointment to a telephone appointment? • What would be a good outcome? • What else needs to happen to make sure a good outcome is achieved? Relates to IPT: Flexibility, convenience, resources* If digital clinical consultations [I] are offered flexibly within a hybrid model [C] it gives women more control over the time, money and effort they have to engage with care [M], and therefore makes it easier for them to access and engage with services [O]** *Notes*: *This scenario relates to a perception that digital clinical consultations are ‘convenient’ because women are not required to travel, take time-off work or organise childcare in order to attend appointments. ** C=Context, M=Mechanism, O=Outcome

The IPTs will be tested, elaborated and refined in the remaining Phases of the review. As the review progresses, a series of further workshops and webinars are planned to continue to engage and consult our stakeholders.

## Conclusion

Overall, the involvement of stakeholders in the first Phase of the review ensured that issues of equity, diversity and inclusion became priorities for the way we focused our enquiry. Initial stakeholder discussions provided context and insights that we had not yet identified from the literature. Later discussions added nuance to our IPTs and gave us confidence that these were on the right track. The mechanisms proposed within the IPTs relate to core aspects of midwifery philosophy and practice, highlighting the importance of integrating these into digitally delivered care.

Innovations in digital health are a rapidly moving field in which the evidence may be theoretically sparse or quickly out of date. Involvement of stakeholders should be an essential component of midwifery- or nurse-led research in this area. Realist programme theories and methods seek to be transferable across similar contexts (Rycroft-Malone et al., 2012). Hence, our work provides valuable insights that can inform future digital nursing and midwifery research.

**Key points for policy, practice and/or research**

- This realist inquiry on digital clinical consultation demonstrates the value of a theory-based approach to delivering digital transformation that can be more widely applied to nurse- and midwifery-led consultation practices.
- Where topics (such as digital consultations) have not yet been heavily theorised, combining diverse evidence sources with the professional and experiential knowledge of stakeholders (including service users and frontline midwives/nurses) is essential for initial programme theory development.
- Nurse and midwife researchers can lead co-production in digital health transformation through the involvement of stakeholders from the very earliest stages of a project.
- The current lack of guidance on DC-CON in maternity care and early stages of the digital transformation makes this an ideal time for nurses/midwives to shape implementation.

## Supplemental Material

sj-pdf-1-jrn-10.1177_17449871241226911 – Supplemental material for Developing initial programme theories for a realist synthesis on digital clinical consultations in maternity care: contributions from stakeholder involvement Supplemental material, sj-pdf-1-jrn-10.1177_17449871241226911 for Developing initial programme theories for a realist synthesis on digital clinical consultations in maternity care: contributions from stakeholder involvement by Catrin Evans, Georgia Clancy, Kerry Evans, Andrew Booth, Benash Nazmeen, Stephen Timmons, Candice Sunney, Mark Clowes, Nia Wyn Jones and Helen Spiby in Journal of Research in Nursing

sj-pdf-2-jrn-10.1177_17449871241226911 – Supplemental material for Developing initial programme theories for a realist synthesis on digital clinical consultations in maternity care: contributions from stakeholder involvement Supplemental material, sj-pdf-2-jrn-10.1177_17449871241226911 for Developing initial programme theories for a realist synthesis on digital clinical consultations in maternity care: contributions from stakeholder involvement by Catrin Evans, Georgia Clancy, Kerry Evans, Andrew Booth, Benash Nazmeen, Stephen Timmons, Candice Sunney, Mark Clowes, Nia Wyn Jones and Helen Spiby in Journal of Research in Nursing

sj-pdf-3-jrn-10.1177_17449871241226911 – Supplemental material for Developing initial programme theories for a realist synthesis on digital clinical consultations in maternity care: contributions from stakeholder involvement Supplemental material, sj-pdf-3-jrn-10.1177_17449871241226911 for Developing initial programme theories for a realist synthesis on digital clinical consultations in maternity care: contributions from stakeholder involvement by Catrin Evans, Georgia Clancy, Kerry Evans, Andrew Booth, Benash Nazmeen, Stephen Timmons, Candice Sunney, Mark Clowes, Nia Wyn Jones and Helen Spiby in Journal of Research in Nursing

sj-pdf-4-jrn-10.1177_17449871241226911 – Supplemental material for Developing initial programme theories for a realist synthesis on digital clinical consultations in maternity care: contributions from stakeholder involvement Supplemental material, sj-pdf-4-jrn-10.1177_17449871241226911 for Developing initial programme theories for a realist synthesis on digital clinical consultations in maternity care: contributions from stakeholder involvement by Catrin Evans, Georgia Clancy, Kerry Evans, Andrew Booth, Benash Nazmeen, Stephen Timmons, Candice Sunney, Mark Clowes, Nia Wyn Jones and Helen Spiby in Journal of Research in Nursing

sj-pdf-5-jrn-10.1177_17449871241226911 – Supplemental material for Developing initial programme theories for a realist synthesis on digital clinical consultations in maternity care: contributions from stakeholder involvement Supplemental material, sj-pdf-5-jrn-10.1177_17449871241226911 for Developing initial programme theories for a realist synthesis on digital clinical consultations in maternity care: contributions from stakeholder involvement by Catrin Evans, Georgia Clancy, Kerry Evans, Andrew Booth, Benash Nazmeen, Stephen Timmons, Candice Sunney, Mark Clowes, Nia Wyn Jones and Helen Spiby in Journal of Research in Nursing

sj-pdf-6-jrn-10.1177_17449871241226911 – Supplemental material for Developing initial programme theories for a realist synthesis on digital clinical consultations in maternity care: contributions from stakeholder involvement Supplemental material, sj-pdf-6-jrn-10.1177_17449871241226911 for Developing initial programme theories for a realist synthesis on digital clinical consultations in maternity care: contributions from stakeholder involvement by Catrin Evans, Georgia Clancy, Kerry Evans, Andrew Booth, Benash Nazmeen, Stephen Timmons, Candice Sunney, Mark Clowes, Nia Wyn Jones and Helen Spiby in Journal of Research in Nursing

sj-pdf-7-jrn-10.1177_17449871241226911 – Supplemental material for Developing initial programme theories for a realist synthesis on digital clinical consultations in maternity care: contributions from stakeholder involvement Supplemental material, sj-pdf-7-jrn-10.1177_17449871241226911 for Developing initial programme theories for a realist synthesis on digital clinical consultations in maternity care: contributions from stakeholder involvement by Catrin Evans, Georgia Clancy, Kerry Evans, Andrew Booth, Benash Nazmeen, Stephen Timmons, Candice Sunney, Mark Clowes, Nia Wyn Jones and Helen Spiby in Journal of Research in Nursing

sj-pdf-8-jrn-10.1177_17449871241226911 – Supplemental material for Developing initial programme theories for a realist synthesis on digital clinical consultations in maternity care: contributions from stakeholder involvement Supplemental material, sj-pdf-8-jrn-10.1177_17449871241226911 for Developing initial programme theories for a realist synthesis on digital clinical consultations in maternity care: contributions from stakeholder involvement by Catrin Evans, Georgia Clancy, Kerry Evans, Andrew Booth, Benash Nazmeen, Stephen Timmons, Candice Sunney, Mark Clowes, Nia Wyn Jones and Helen Spiby in Journal of Research in Nursing
