# Supplementary material for: Developing initial programme theories for a realist synthesis on digital clinical consultations in maternity care: contributions from stakeholder involvement
Source: J Res Nurs. 2024 Mar 8;29(2):127–40. doi: 10.1177/17449871241226911 (PMC11271666; doi:10.1177/17449871241226911)
Supplement: sj-pdf-3-jrn-10.1177_17449871241226911 – Supplemental material for Developing initial programme theories for a realist synthesis on digital clinical consultations in maternity care: contributions from stakeholder involvement [file sj-pdf-3-jrn-10.1177_17449871241226911.pdf]

Supplementary File S3: Phase One Initial Database Search Strategy

Database: Ovid MEDLINE(R) and Epub Ahead of Print, In-Process, In-Data-Review & Other Non-Indexed Citations, Daily and Versions(R) <1946 to January 27, 2022>

#	Query	Results from 28 Jan 2022
1	exp Telemedicine/	38,848
2	remote consultation/ or videoconferencing/	7,285
3	(telemedicine or tele-medicine or telecare or tele-care or telehealth or tele-health).mp. [mp=title, abstract, original title, name of substance word, subject heading word, floating sub-heading word, keyword heading word, organism supplementary concept word, protocol supplementary concept word, rare disease supplementary concept word, unique identifier, synonyms]	44,080
4	((remote* or virtual* or online or on-line or digital*) adj3 (consultation* or appointment* or meet*)).mp.	8,514
5	(videoconferenc* or video-conferenc* or teleconferenc* or tele-conferenc* or zoom or facetime or face-time).mp.	8,842
6	1 or 2 or 3 or 4 or 5	56,928
7	exp Maternal Health Services/	54,815
8	exp Prenatal Care/ or exp Midwifery/ or exp Pregnancy/ or exp Obstetrics/	972,055
9	(matern* or pregnan* or prenatal or pre-natal or antenatal or ante-natal or perinatal or peri-natal or postnatal or post-natal or postpartum or post-partum or breastfeed* or breast feed* or midwi* or obstetric*).mp.	1,408,667
10	7 or 8 or 9	1,420,532
11	6 and 10	1,835
12	Primary Health Care/	86,523
13	general practice/ or family practice/	77,081
14	(primary care or general practice or GP or family doctor* or family physician*).mp.	225,104
15	exp Endocrinologists/	222
16	exp Cardiologists/	986
17	(endocrinologist* or cardiologist*).mp.	21,716
18	12 or 13 or 14 or 15 or 16 or 17	309,535
19	6 and 18	4,484
20	19 not 11	4,360
21	(framework* or model* or theor* or concept*).mp.	5,371,555
22	limit 21 to yr="2010 -Current"	3,213,643
23	11 and 22	407
24	20 and 22	1,060
