# Supplementary material for: Developing initial programme theories for a realist synthesis on digital clinical consultations in maternity care: contributions from stakeholder involvement
Source: J Res Nurs. 2024 Mar 8;29(2):127–40. doi: 10.1177/17449871241226911 (PMC11271666; doi:10.1177/17449871241226911)
Supplement: sj-pdf-4-jrn-10.1177_17449871241226911 – Supplemental material for Developing initial programme theories for a realist synthesis on digital clinical consultations in maternity care: contributions from stakeholder involvement [file sj-pdf-4-jrn-10.1177_17449871241226911.pdf]

Supplementary File S4: Phase One Sampling Framework/Evidence Sources

Context/Relevance	Sample and References	Total Number of Documents/Papers
**Empirical Papers and Reviews: Maternity context**	• Mixed/general (Penny et al., 2018)<br>• Antenatal (Tavener et al., 2022; Wu et al., 2021; Reid et al., 2021; Hinton et al., 2022)<br>• Postnatal (Saad et al., 2021)<br>• Infant feeding (Habibi et al., 2012)<br>• Early labour (Faucher and Kennedy, 2020; Spiby et al., 2019)<br>• High risk/monitoring (van den Heuvel et al., 2020; Khalil, 2019)<br>• Migrant women/language issues (Rayment-Jones et al., 2021)<br>• Black and ethnic minority women (John et al., 2021; Pilav et al., 2022)<br>• Mental health concerns (Pilav et al., 2022)<br>• Social risk factors (Rayment-Jones et al., 2019)<br>• Low resource setting (Kabongo et al., 2021; Abejirinde et al., 2018)	n=17
**Empirical Papers and Reviews: Non-maternity Context**	• Existing (related) realist reviews (Huxley et al., 2015; Vassilev et al., 2015)<br>• Primary care (Murphy et al., 2021)<br>• Mental health (Liberati et al., 2022; Greenhalgh and Wherton, 2022)<br>• Rehabilitation (Gilbert et al., 2021; Gilbert et al., 2022)<br>• Young people (Griffiths et al., 2017)<br>• General (related) review(Mann et al., 2021)	n=9
**Policy, Guidance, Opinion**	• RCM, RCN, RCOG Guidance(Royal College of Midwives, 2021a; Royal College of Midwives, 2021b; Royal College of Midwives and Royal College of Obstetricians & Gynaecologists, 2020; Royal College of Nursing, 2020; Royal College of Obstetricians & Gynaecologists, 2020)<br>• NHS RHO Report (Kapadia et al., 2022)<br>• Maternity Transformation (NHS England)<br>• Digital Maternity Reports (NHS Digital; NHS Digital, 2018)<br>• Commentaries on remote maternity care (Hinton et al., 2021; Kuberska et al., 2021)	n=11
**Frameworks and Theories (and Select Associated Exemplar Papers)**	• Conceptual Framework - Planning and Evaluation of Remote Consultation Services (PERCS) (Greenhalgh and Wherton, 2022; Greenhalgh et al., 2021; Greenhalgh et al., 2022; Shaw et al., 2021)<br>• Candidacy Theory (Dixon-Woods et al., 2006; Mackintosh et al., 2021; Liberati et al., 2021)<br>• Burden of Treatment Theory (Gilbert et al., 2021; Gallacher et al., 2018; Mair and May, 2014; Mair et al., 2021; May et al., 2009; May et al., 2014)<br>• Normalisation Process Theory (Gilbert et al., 2022; Murphy et al., 2021; May et al., 2020)	n=16 (of which n=4 are also in the categories above)

References of Included Papers

Abejirinde IO, Ilozumba O, Marchal B, et al. (2018) Mobile health and the performance of maternal health care workers in low- and middle-income countries: A realist review. *Int J Care Coord* 21(3): 73-86.

Dixon-Woods M, Cavers D, Agarwal S, et al. (2006) Conducting a critical interpretive synthesis of the literature on access to healthcare by vulnerable groups. *BMC Med Res Methodol* 6: 35.

Faucher MA and Kennedy HP (2020) Women's Perceptions on the Use of Video Technology in Early Labor: Being Able to See. *Journal of midwifery & women's health* 65(3): 342-348.

Gallacher KI, May CR, Langhorne P, et al. (2018) A conceptual model of treatment burden and patient capacity in stroke. *BMC Fam Pract* 19(1): 9.
Gilbert AW, Jones J, Stokes M, et al. (2021) Factors that influence patient preferences for virtual consultations in an orthopaedic rehabilitation setting: a qualitative study. *BMJ open* 11(2): e041038.
Gilbert AW, Jones J, Stokes M, et al. (2022) Patient, clinician and manager experience of the accelerated implementation of virtual consultations following COVID-19: A qualitative study of preferences in a tertiary orthopaedic rehabilitation setting. *Health Expect*. Epub ahead of print 2022/01/12. DOI: 10.1111/hex.13425.
Greenhalgh T, Ladds E, Hughes G, et al. (2022) Why do GPs rarely do video consultations? qualitative study in UK general practice. *British Journal of General Practice*. DOI: 10.3399/BJGP.2021.0658. BJGP.2021.0658.
Greenhalgh T, Rosen R, Shaw SE, et al. (2021) Planning and Evaluating Remote Consultation Services: A New Conceptual Framework Incorporating Complexity and Practical Ethics. *Front Digit Health* 3: 726095.
Greenhalgh T and Wherton J (2022) Telepsychiatry: learning from the pandemic. *The British Journal of Psychiatry*. Epub ahead of print 2022/02/18. DOI: 10.1192/bjp.2021.224. 1-5.
Griffiths F, Bryce C, Cave J, et al. (2017) Timely Digital Patient-Clinician Communication in Specialist Clinical Services for Young People: A Mixed-Methods Study (The LYNC Study). *Journal of medical Internet research* 19(4): e102-e102.
Habibi MF, Nicklas J, Spence M, et al. (2012) Remote lactation consultation: a qualitative study of maternal response to experience and recommendations for survey development. *J Hum Lact* 28(2): 211-217.
Hinton L, Dakin FH, Kuberska K, et al. (2022) Quality framework for remote antenatal care: qualitative study with women, healthcare professionals and system-level stakeholders. *BMJ quality & safety*. DOI: 10.1136/bmjqs-2021-014329.
Hinton L, Kuberska K, Dakin F, et al. (2021) Creating equitable remote antenatal care: the importance of inclusion. *BMJ Opinion*.
Huxley CJ, Atherton H, Watkins JA, et al. (2015) Digital communication between clinician and patient and the impact on marginalised groups: a realist review in general practice. *British Journal of General Practice* 65(641): e813.
John JR, Curry G and Cunningham-Burley S (2021) Exploring ethnic minority women's experiences of maternity care during the SARS-CoV-2 pandemic: a qualitative study. *BMJ open* 11(9): e050666.
Kabongo EM, Mukumbang FC, Delobelle P, et al. (2021) Explaining the impact of mHealth on maternal and child health care in low- and middle-income countries: a realist synthesis. *BMC pregnancy and childbirth* 21(1): 196.
Kapadia D, Zhang J, Salway S, et al. (2022) Ethnic Inequalities in Healthcare: A Rapid Evidence Review. Reportno. Report Number|, Date. Place Published|: Institution|.
Khalil C (2019) Understanding the Adoption and Diffusion of a Telemonitoring Solution in Gestational Diabetes Mellitus: Qualitative Study. *JMIR diabetes* 4(4): e13661.
Kuberska K, Dakin F, Dixon-Woods M, et al. (2021) Creating an equitable evidence base for quality and safety in remote antenatal care. *Authorea (Pre-Print)*. DOI: 10.22541/au.160861376.62206303/v1.
Liberati E, Richards N, Parker J, et al. (2021) Remote care for mental health: qualitative study with service users, carers and staff during the COVID-19 pandemic. *BMJ open* 11(4): e049210.
Liberati E, Richards N, Parker J, et al. (2022) Qualitative study of candidacy and access to secondary mental health services during the COVID-19 pandemic. *Social Science & Medicine* 296: 114711.
Mackintosh N, Gong QS, Hadjiconstantinou M, et al. (2021) Digital mediation of candidacy in maternity care: Managing boundaries between physiology and pathology. *Soc Sci Med* 285: 114299.
Mair FS and May CR (2014) Thinking about the burden of treatment. *BMJ* 349: g6680.
Mair FS, Montori VM and May CR (2021) Digital transformation could increase the burden of treatment on patients. *BMJ* 375: n2909.
Mann C, Turner A and Salisbury C (2021) The impact of remote consultations on personalised care: Evidence briefing (Commisssioned by the Personalised Care Institute). Reportno. Report Number|, Date. Place Published|: Institution|.
May C, Finch T and Rapley T (2020) Normalization Process Theory (Chapter 6). In: Nilsen P and Birken S (eds) *Handbook on Implementation Science* Edward Elgar Publishing Ltd, pp.144-167.
May C, Montori VM and Mair FS (2009) We need minimally disruptive medicine. *BMJ* 339: b2803.
May CR, Eton DT, Boehmer K, et al. (2014) Rethinking the patient: using Burden of Treatment Theory to understand the changing dynamics of illness. *BMC health services research* 14(1): 281.
Murphy M, Scott LJ, Salisbury C, et al. (2021) Implementation of remote consulting in UK primary care following the COVID-19 pandemic: a mixed-methods longitudinal study. *The British Journal of General Practice* 71(704): e166-e177.
NHS Digital Digital Maternity: Harnessing Digital Technology in Maternity Services. Reportno. Report Number|, Date. Place Published|: Institution|.
NHS Digital (2018) Maternity DMA Report: Digital Maturity Assessment of Maternity Services in England. Reportno. Report Number|, Date. Place Published|: Institution|.
NHS England Maternity Transformation Programme. Reportno. Report Number|, Date. Place Published|: Institution|.

Penny RA, Bradford NK and Langbecker D (2018) Registered nurse and midwife experiences of using videoconferencing in practice: A systematic review of qualitative studies. *Journal of clinical nursing* 27(5-6): e739-e752.

Pilav S, Easter A, Silverio SA, et al. (2022) Experiences of Perinatal Mental Health Care among Minority Ethnic Women during the COVID-19 Pandemic in London: A Qualitative Study. *Int J Environ Res Public Health* 19(4).

Rayment-Jones H, Harris J, Harden A, et al. (2019) How do women with social risk factors experience United Kingdom maternity care? A realist synthesis. *Birth* 46(3): 461-474.

Rayment-Jones H, Harris J, Harden A, et al. (2021) Project20: interpreter services for pregnant women with social risk factors in England: what works, for whom, in what circumstances, and how? *Int J Equity Health* 20(1): 233.

Reid CN, Marshall J and Fryer K (2021) Evaluation of a Rapid Implementation of Telemedicine for Delivery of Obstetric Care During the COVID-19 Pandemic. *medRxiv*. DOI: 10.1101/2021.05.19.21257311. 2021.2005.2019.21257311.

Royal College of Midwives (2021a) Digital Technology in Maternity Care: A Position Statement. Reportno. Report Number|, Date. Place Published|: Institution|.

Royal College of Midwives (2021b) Virtual Consultations: Guidance on Appropriate Application for Virtual Consultations and Practical Tips for Effective Use. Reportno. Report Number|, Date. Place Published|: Institution|.

Royal College of Midwives and Royal College of Obstetricians & Gynaecologists (2020) Guidance for Antenatal and Postnatal Services in the evolving Coronavirus (COVID-19) pandemic (Version 3). Reportno. Report Number|, Date. Place Published|: Institution|.

Royal College of Nursing (2020) Remote consultations guidance under COVID-19 restrictions. Reportno. Report Number|, Date. Place Published|: Institution|.

Royal College of Obstetricians & Gynaecologists (2020) Self Monitoring of Blood Pressure in Pregnancy: Information for Healthcare Professionals, Version 1. Reportno. Report Number|, Date. Place Published|: Institution|.

Saad M, Chan S, Nguyen L, et al. (2021) Patient perceptions of the benefits and barriers of virtual postnatal care: a qualitative study. *BMC pregnancy and childbirth* 21(1): 543-543.

Shaw SE, Hughes G, Wherton J, et al. (2021) Achieving Spread, Scale Up and Sustainability of Video Consulting Services During the COVID-19 Pandemic? Findings From a Comparative Case Study of Policy Implementation in England, Wales, Scotland and Northern Ireland. *Frontiers in Digital Health* 3.

Spiby H, Faucher MA, Sands G, et al. (2019) A qualitative study of midwives' perceptions on using video-calling in early labor. *Birth* 46(1): 105-112.

Tavener CR, Kyriacou C, Elmascri I, et al. (2022) Rapid introduction of virtual consultation in a hospital-based Consultant-led Antenatal Clinic to minimise exposure of pregnant women to COVID-19. *BMJ open quality* 11(1): e001622.

van den Heuvel JFM, Teunis CJ, Franx A, et al. (2020) Home-based telemonitoring versus hospital admission in high risk pregnancies: a qualitative study on women’s experiences. *BMC pregnancy and childbirth* 20(1): 77.

Vassilev I, Rowsell A, Pope C, et al. (2015) Assessing the implementability of telehealth interventions for self-management support: a realist review. *Implement Sci* 10: 59.

Wu K, Lopez C and Nichols M (2021) Virtual Visits in Prenatal Care: An Integrative Review. *Journal of midwifery & women's health*. DOI: https://dx.doi.org/10.1111/jmwh.13284.
