# Supplementary material for: Developing initial programme theories for a realist synthesis on digital clinical consultations in maternity care: contributions from stakeholder involvement
Source: J Res Nurs. 2024 Mar 8;29(2):127–40. doi: 10.1177/17449871241226911 (PMC11271666; doi:10.1177/17449871241226911)
Supplement: sj-pdf-5-jrn-10.1177_17449871241226911 – Supplemental material for Developing initial programme theories for a realist synthesis on digital clinical consultations in maternity care: contributions from stakeholder involvement [file sj-pdf-5-jrn-10.1177_17449871241226911.pdf]

Supplementary File S5: Phase One Appraisal and Data Extraction Form

Research Question [Reminder]

How can digital clinical consultations be implemented in a clinically safe, appropriate and acceptable way in maternity care in the UK NHS? For whom? In what settings? And for what purposes?

Reference

Insert study reference

Extraction Questions

- What is this paper about?
- Does it include data relevant to IPT development?
- What are the key casual insights and explanations in this paper?
  - If possible, express these as an If-Then statement or in the form of a CMO configuration (Context-Mechanism-Outcome).
  - If it is not very clear how to do this, express the key insights or causal explanations in your own words
- Are the key insights directly (or indirectly) linked to outcomes?
  - Can you specify the outcomes and, if possible, explain how they are linked? (Ignore this question if you have already included this information above)
- Is there anything surprising or unexpected in this paper? Briefly summarise your thoughts
- Are any of the key insights different to what we have read in other papers or heard in the stakeholder groups? Why might this be the case?
