# Supplementary material for: Developing initial programme theories for a realist synthesis on digital clinical consultations in maternity care: contributions from stakeholder involvement
Source: J Res Nurs. 2024 Mar 8;29(2):127–40. doi: 10.1177/17449871241226911 (PMC11271666; doi:10.1177/17449871241226911)
Supplement: sj-pdf-6-jrn-10.1177_17449871241226911 – Supplemental material for Developing initial programme theories for a realist synthesis on digital clinical consultations in maternity care: contributions from stakeholder involvement [file sj-pdf-6-jrn-10.1177_17449871241226911.pdf]

Supplementary File S6: Phase One Mid-Range Theory Extraction and Analysis Form

Original Theory Paper

Reference

Exemplar Paper(s) Applied to DC-CON

Reference

Definition/Summary of the Theory

Definition

Core Concepts of the Theory (from theory paper)	Application to DC-CON (from exemplar paper)	Potential Application to DC-CON in Maternity Care (using abductive reasoning)
