# Supplementary material for: Developing initial programme theories for a realist synthesis on digital clinical consultations in maternity care: contributions from stakeholder involvement
Source: J Res Nurs. 2024 Mar 8;29(2):127–40. doi: 10.1177/17449871241226911 (PMC11271666; doi:10.1177/17449871241226911)
Supplement: sj-pdf-7-jrn-10.1177_17449871241226911 – Supplemental material for Developing initial programme theories for a realist synthesis on digital clinical consultations in maternity care: contributions from stakeholder involvement [file sj-pdf-7-jrn-10.1177_17449871241226911.pdf]

Supplementary File S7: Initial Programme Theories

Domain	IPT
**Organisation**	
**Resourcing of Infrastructure and Support**	1. *If organisations provide a well-resourced digital infrastructure [**C**] (including reliable equipment, software, internet), staff will feel confident [**M**] that digital consultations [**I**] are a tool that can 'fit' into existing work practices [**C**]. Hence, staff will feel motivated [**M**] to embed it into their practice [**O**].*
	2. *If NHS and professional organisations provide a supportive and enabling workplace culture for digital clinical consultations [**I**] (including sufficient training and access to clinical, technical and administrative support) [**C**], staff will gain relevant knowledge/skills [**M**] and will feel motivated, supported and confident [**M**], leading to appropriate and sustained uptake of digital consultations [**O**].*
**Clinical Guidance and Integration**	3. *If digital consultations [**I**] are supported by administrative systems and guided by clear clinical protocols and staffing models which can be adapted to meet women's varied needs [**C**], staff will feel confident, supported and motivated [**M**] to use it (and sustain its use). This is because it will be perceived to improve existing workflows - increasing convenience and efficiency and reducing workload [**O**] – for themselves and service users as well as maintaining safety [**O**].*
	4. *If digital clinical consultations are integrated into electronic patient record systems that can operate across contexts [**C**], this will improve the ability of staff to access information, communicate with multiple stakeholders and coordinate care across the care pathway [**M**]. This enhances staff ability to monitor, support and treat women [**O**], leading to job satisfaction and optimal clinical/safety outcomes [**O**].*
**Healthcare Professionals**	
**Perceived Benefit and Staff Motivation**	5. *If staff perceive [**M**] that women are benefitting from, and satisfied [**O**] with, digital consultations [**I**] they will be motivated [**M**] to use it (buy into and sustain its use) [**O**] and gain job satisfaction from using it [**O**].*
**Professional Autonomy in Mode of Consultation**	6. *Digital clinical consultations [**I**] provide staff with additional methods with which to communicate with women [**C**]. Staff appreciate having the professional autonomy [**M**] to select the most appropriate mode of consultation [**I**] to meet the woman's needs as well as their own workload [**C**]. This enables staff to work flexibly [**M**] and empowers [**M**] them to personalise care [**M**] and develop relationships with women [**M**], leading to job satisfaction, optimal clinical outcomes and staff 'buy-in' to the sustained use [**O**] of digital consultations [**I**].*
	*7. When staff are matching the mode of consultation to the reason for consultation [**C**], the convenience and flexibility [**M**] of the telephone is typically considered most suitable for routine and transactional care [**C**], as it promotes efficiency [**O**]. However, the option and ability to visualise a service user (e.g. either through video or face to face) [**C**] gives staff additional confidence and control in managing uncertainty [**M**], especially where there is a concern [**M**] to identify and manage any physical, psychological or social risks [**C**] - leading to optimal clinical outcomes [**O**] and safety assurances.*

Domain	IPT
**Communication and Relational Care**	8. *For both routine and more complex care situations [**C**], staff and women feel more able to communicate openly and to be understood [**M**] if a pre-existing trusting relationship [**C**] has been established, leading to satisfaction and optimal clinical outcomes [**O**].*
**Women and Families**	
**Knowledge, Choice and Fit with Preferences**	9. *If women are made aware of, and informed about, the different types of consultations [**I**] available to them [**M**], this will empower women [**M**] to make informed choices about the mode of care they receive [**M**] and improve the potential for personalisation [**M**] of care delivery which often improves clinical and social outcomes [**O**]. However, if women's preferences and expectations of care are in alignment with the digital consultation modality offered by their healthcare provider [**C**] then women are likely to be satisfied with the service provided [**O**], regardless of whether or not the care was personalised or women actively made choices about the mode of care [**M**].*
**Navigation and Inclusion**	10. *Whilst there can be benefits to using digital clinical consultations [**I**], for women who face language [**C**] or other communication barriers [**C**], or who lack financial/digital resources [**C**], digital clinical consultations [**I**] can present a challenge to accessing care [**O**] leading to a lack of knowledge about services [**M**], creating frustration or anxiety and a lack of motivation or sense of entitlement [**M**] to engage with care [**O**]. This increases inequalities [**O**] and can lead to women not making informed choices, important issues being missed and sub-optimal clinical outcomes [**O**].*
**Control of Resources**	11. *If digital consultations [**I**] are easy to use [**M**] and fit flexibly [**M**] with women's preferences, life circumstances and clinical needs [**C**], it gives them more control over the time, money and effort they have to engage with care [**M**] which can be a relief [**M**] and for some women will make it less burdensome [**M**] for them to access and engage with services [**O**].*
**Personalisation, Connection and Support**	12. *If digital consultations [**I**] are personalised [**M**] to women's needs, preferences and life circumstances [**C**], women feel a sense of safety, reassurance and empowerment [**M**] through an enhanced sense of connection to services and staff [**M**] (these benefits may be dependent on the healthcare provider-woman relationship [**C**], a maternity record system that supports communication and a system for digital consultations [**I**]). This leads to increased self-efficacy and motivation [**M**] contributing to satisfaction, empowerment and optimal clinical outcomes [**O**].*
**Empowerment and Involvement**	13. *If women have the capability to use digital consultations [**C**], it can make it easier to include women's partners [**M**] and facilitate women's active participation [**M**] alongside their healthcare provider(s). This can empower, motivate and give women a sense of control over their health and care, [**M**] improving access and enhancing engagement with services [**O**].*
