# Supplementary material for: Developing initial programme theories for a realist synthesis on digital clinical consultations in maternity care: contributions from stakeholder involvement
Source: J Res Nurs. 2024 Mar 8;29(2):127–40. doi: 10.1177/17449871241226911 (PMC11271666; doi:10.1177/17449871241226911)
Supplement: sj-pdf-8-jrn-10.1177_17449871241226911 – Supplemental material for Developing initial programme theories for a realist synthesis on digital clinical consultations in maternity care: contributions from stakeholder involvement [file sj-pdf-8-jrn-10.1177_17449871241226911.pdf]

Supplementary File S8: Scenarios with Embedded Women-Focused Initial Programme Theories

These need to be read in conjunction with Supplementary File 6 containing the Table of Initial Programme Theories (IPTs).

The IPTs related to women's experiences were incorporated into 4 different scenarios. These are described below, alongside some explanatory notes. For each one, there is a short scenario, followed by questions that were posed to the Community and Service User Stakeholder Group (COSU-SG).

Scenario 1

Jade works part-time, with a toddler and a baby on the way. She has an upcoming appointment to discuss her birth plan with her midwife at the birth centre. However, Jade's car is currently at the garage having work done and the birth centre is in the next town. This means Jade would have to take three different buses, with her toddler, in order to attend the appointment. The journey will also take much longer on the bus so she might have to take time off work. Concerned about this, Jade rang the birth centre where the staff suggested that they change her appointment to a telephone consultation.

<u>Questions for the Group</u>

- How do you think Jade would feel about the suggestion to change her face-to-face appointment to a telephone appointment?
- What would be a good outcome?
- What else needs to happen to make sure a good outcome is achieved?

Relates to IPT: 'Flexibility, convenience, resources'*:*
If digital clinical consultations [I] are offered flexibly within a hybrid model [C] it gives women more control over the time, money and effort they have to engage with care [M], and therefore makes it easier for them to access and engage with services [O].

Notes: This relates to the common perception that digital clinical consultations are 'convenient' because women are not required to travel, take time-off work or organise childcare in order to attend appointments.

Scenario 2

Alice is pregnant with her first child. She is in her final trimester and still working full-time. Recently she has been struggling with pelvic pain which is making it difficult to move about. During her lunch break she gave the maternity triage line a call. The midwife she spoke to could see from Alice's maternity record that she had called several times about this issue and was feeling very anxious about the pregnancy. They booked Alice a video consultation that evening with a midwife she had met before. Alice was told that she would be emailed instructions on how to access the video consultation before the appointment.

<u>Questions for the Group</u>

- How do you think Alice would feel about the video consultation that evening?
- What impact, if any, do you think Alice having met the midwife before will have on the video consultation?
- What would be a good outcome?

Relates to IPT: 'Personalisation and reassurance':
If remote care is personalised [C] to women's needs and life circumstances [C], women feel a sense of safety, reassurance and empowerment [M] through an enhanced sense of connection to services and staff [M] (these benefits may be dependent on the healthcare provider-woman relationship [C] and a maternity record system that supports communication [I]). This leads to increased self-efficacy and motivation [M] contributing to satisfaction, empowerment and optimal clinical outcomes [O].

Notes: This relates to the possibility that digital consultations can more easily be accessed on an ad hoc basis than face-to-face appointments, giving women care/reassurance when they need it.

Scenario 3

Zainab has developed gestational diabetes during pregnancy. To help monitor her condition Zainab was given a blood sugar testing kit to use at home. This kit includes a monitor which connects wirelessly to an App on Zainab's smartphone and allows her to easily send her blood sugar readings to her midwife and obstetrician. In a recent video consultation with Zainab and her partner, her obstetrician explained how the monitoring App works, what her recent blood sugar readings meant and what she could do to make sure they stay within a normal range.

Questions for the Group
- How do you think Zainab would feel about the at-home monitoring kit and App?
  - Do you think it would affect her behaviour?
- What impact do you think the use of remote consultations has on Zainab's maternity experience?
- How do you think remote care might facilitate the involvement of different professionals in Zainab's care?

Relates to IPT: 'Empowerment and family involvement':
If the system and process [C] of using digital clinical consultations [I] is clear [C], fits easily [C] into women's lives [C] and can facilitate women's active participation [M], as well as the potential to include partners/families [M], then women will be empowered and motivated to use it [M]. This can improve access and enhance engagement with services [O].

Notes: This relates to the technological logistics involved in attending a digital consultation, e.g. the provision of instructions and devices/software required .'Active participation' refers to the incorporation of at-home monitoring tools into digital clinical consultations and the potential for healthcare providers to share visual data with women during a digital consultation.

Scenario 4
Karolina has recently moved to England from Poland and although she hasn't used the NHS much since she's been here, she has now found out that she is pregnant. A friend advised her that she should call her local maternity unit and arrange a 'booking in' appointment. Whilst Karolina does speak some English, when she called the maternity unit she mentioned that she was nervous about the booking in appointment in case there was a lot of technical language that she didn't understand. The staff informed her that the booking in appointment would be a digital consultation and she could choose either a telephone or video call.

Questions for the Group
- Is being able to choose between a telephone or video call important? – Why?
- Having recently moved to England, there is a sense that Karolina doesn't know much about the NHS, why does this matter?

- What impact might it have on her maternity experience if Karolina doesn't understand technical birth language?
- Who needs to be involved in Karolina's appointment to help achieve a good outcome?

Relates to IPT: 'Communication barriers and digital inclusion':
Whilst there can be benefits to using digital clinical consultations, for women who face language [C] or other communication barriers [C], or who lack financial/digital resources [C], digital clinical consultations [I] can present a challenge to accessing care [O] leading to a lack of knowledge about services [M], creating frustration or anxiety and a lack of motivation or sense of entitlement [M] to engage with care [O]. This increases inequalities [O] and can lead to important issues being missed and sub-optimal clinical outcomes [O].

Notes: This relates to the need to have systems in place which can help to support those who might find digital consultations particularly challenging. Language or communication barriers refers to those for whom English is not their first language as well as those with visual/hearing impairments and learning difficulties.
